# Ultrasound evidence of altered lumbar connective tissue structure in human subjects with chronic low back pain

**DOI:** 10.1186/1471-2474-10-151

**Published:** 2009-12-03

**Authors:** Helene M Langevin, Debbie Stevens-Tuttle, James R Fox, Gary J Badger, Nicole A Bouffard, Martin H Krag, Junru Wu, Sharon M Henry

**Affiliations:** 1 Departments of Neurology, Given Building, University of Vermont, Burlington VT 05405, USA; 2 Department of Orthopaedics & Rehabilitation, Stafford Building, University of Vermont, Burlington VT 05405, USA; 3 Department of Medical Biostatistics, Hills Building, University of Vermont, Burlington VT 05405, USA; 4 Department of Physics, Cook Building, University of Vermont, Burlington VT 05405, USA; 5 Department of Rehabilitation & Movement Science, Rowell Building, University of Vermont, Burlington VT 05405, USA

## Abstract

**Background:**

Although the connective tissues forming the fascial planes of the back have been hypothesized to play a role in the pathogenesis of chronic low back pain (LBP), there have been no previous studies quantitatively evaluating connective tissue structure in this condition. The goal of this study was to perform an ultrasound-based comparison of perimuscular connective tissue structure in the lumbar region in a group of human subjects with chronic or recurrent LBP for more than 12 months, compared with a group of subjects without LBP.

**Methods:**

In each of 107 human subjects (60 with LBP and 47 without LBP), parasagittal ultrasound images were acquired bilaterally centered on a point 2 cm lateral to the midpoint of the L2-3 interspinous ligament. The outcome measures based on these images were subcutaneous and perimuscular connective tissue thickness and echogenicity measured by ultrasound.

**Results:**

There were no significant differences in age, sex, body mass index (BMI) or activity levels between LBP and No-LBP groups. Perimuscular thickness and echogenicity were not correlated with age but were positively correlated with BMI. The LBP group had ~25% greater perimuscular thickness and echogenicity compared with the No-LBP group (ANCOVA adjusted for BMI, p < 0.01 and p < 0.001 respectively).

**Conclusion:**

This is the first report of abnormal connective tissue structure in the lumbar region in a group of subjects with chronic or recurrent LBP. This finding was not attributable to differences in age, sex, BMI or activity level between groups. Possible causes include genetic factors, abnormal movement patterns and chronic inflammation.

## Background

Chronic low back pain (LBP) is a poorly understood condition causing substantial disability and health care costs worldwide [1-4]. To date, efforts to understand the pathophysiological mechanisms leading to chronic LBP have chiefly focused on structural pathology of the vertebrae and associated tissues [5], neuropsychosocial factors [6-10] and abnormalities of motor control [11-16]. In contrast, the non-specialized connective tissues forming the fascial planes of the back have received little attention. Tearing, inflammation, fibrosis, adhesions, fatty infiltration and herniation within the lumbodorsal fascia have been described in sporadic case reports and series of patients undergoing surgery for LBP since the 1950s [17-27]. Also, several investigators have proposed that fasciae and non-specialized connective tissues could be involved in the pathophysiology of LBP [28-31]. A plausible pathophysiological mechanism is that ongoing local tissue inflammation combined with pain-related movement abnormalities may lead to connective tissue fibrosis, increased tissue stiffness and further movement impairment which may contribute to LBP chronicity [28]. To date, however, no quantitative evaluation of the non-specialized connective tissues of the back in LBP has been reported.

Ultrasound imaging is a non-invasive method that allows visualization of anatomical structures based on reflected ultrasonic waves from interfaces of heterogeneous tissues. We have previously shown that ultrasound imaging can be used to quantitatively evaluate the structure of subcutaneous and perimuscular connective tissue in humans [32]. The goal of the current study was to use ultrasound imaging to compare the structure of connective tissue in the lumbar region of a group of human subjects with LBP compared with a control group of subjects without LBP (No-LBP). We propose that, in chronic LBP, the connective tissues of the back are thicker and more disorganized as a result of remodeling, chronic inflammation, fibrosis and/or fatty infiltration. We therefore tested the hypothesis that, on average, connective tissue thickness and ultrasound echogenicity in the lumbar region is increased in LBP compared with No-LBP.

## Methods

### Human subject recruitment and selection criteria

The study was approved by the University of Vermont Institutional Review Board (CHRMS 07-025) and in compliance with the Helsinki Declaration. All subjects provided informed consent. One hundred and seven human subjects were recruited by advertisements at the University of Vermont and associated facilities. The inclusion criterion for the LBP group was a history of recurrent or chronic LBP for at least 12 months as defined by Von Korff [33,34]. Recurrent LBP was defined as low back pain present on less than half the days in a 12-month period, occurring in multiple episodes over a year. Chronic LBP was defined as back pain present on at least half the days in a 12-month period in a single episode. Inclusion criteria for No-LBP subjects were the absence of a history of low back pain or any other chronic pain that had limited activities of daily living or work and a current numerical current pain index of less than 0.5 (on an 10 point Visual Analogue Scale). Additional exclusion criteria based on a subject's self report for both groups were: previous severe back or low extremity injury or surgery; major structural spinal deformity (scoliosis, kyphosis, stenosis); ankylosing spondylitis or rheumatoid arthritis; spinal fracture, tumor or infection; clinical neurological deficit suggesting nerve root compression; neurological or major psychiatric disorder; bleeding disorders; corticosteroid medication or corticosteroid injection at L2-3 level of the back; pregnancy; worker's compensation or disability case; litigation for LBP; acute systemic infection. Subjects in the LBP group completed the McGill Pain questionnaire [35], the Oswestry Disability Scale questionnaire [36], as well as a custom-designed questionnaire about the onset, history and duration of their LBP. In addition, both groups completed a physical activity level questionnaire in which physical activity level was categorized into sedentary (physical activity less than 3 times per week [< 1 hour cumulative time]), moderate (physical activity 3-5 times per week [1.5 to 3 hours]) and high levels (greater than 5 times per week [> 3 hours]). Subjects with No-LBP were frequency-matched to subjects with LBP for age, sex and BMI in order for the two groups to be balanced for these characteristics.

### Ultrasound image data acquisition

**Figure 1:**
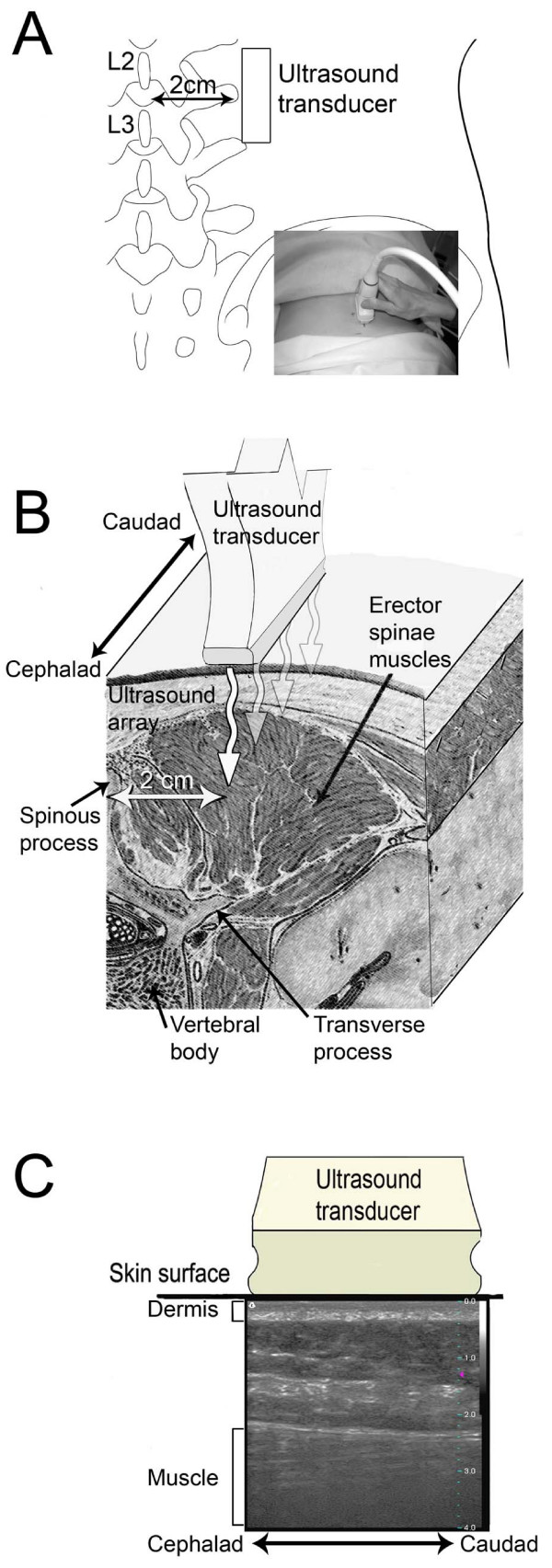
**Ultrasound image acquisition method**. A: location of ultrasound transducer relative to spine; B: anatomical cross-section showing structures imaged by ultrasound beam; C: example of parasagital ultrasound image showing location of dermis and muscle.

Ultrasound images were acquired by an investigator blind to the study condition (LBP vs. No-LBP). Each subject underwent a single testing session consisting of ultrasound B-mode imaging of the lumbar region using a Terason 3000 scanner (Terason, Burlington MA) with a 4 cm, 10 MHz linear array transducer. The location of the paraspinal muscle was first identified during live ultrasound imaging, and the ultrasound focal region was adjusted to be near the superficial border of the muscle. A parasagital image was acquired bilaterally with the transducer centered on a point 2 cm lateral to the middle of the L2-3 interspinous ligament (Figure 1). In preliminary testing, we compared parasagital ultrasound images taken at a constant location (2 cm from the midline) vs. a proportional location (at the midpoint between the midline and the lateral border of the longissimus muscle); measurements of perimuscular connective tissue thickness at those two locations were highly correlated across the two methods (r = 0.99) and there was no evidence of systematic differences between measurements (paired t-test p = 0.71). We therefore chose the constant 2 cm distance for greater method standardization. We chose the L2-3 level in this study because, at this location, the fascia planes are the most parallel to the skin. Further caudal, such as the L4-5 level, the subcutaneous gluteal fat pad begins and causes more variability in the angle between the skin surface and the thoracolumbar fascia. Great care was taken to apply minimal compression on the tissues during ultrasound image acquisition.

### Ultrasound data analysis and echogenicity determination

**Figure 2:**
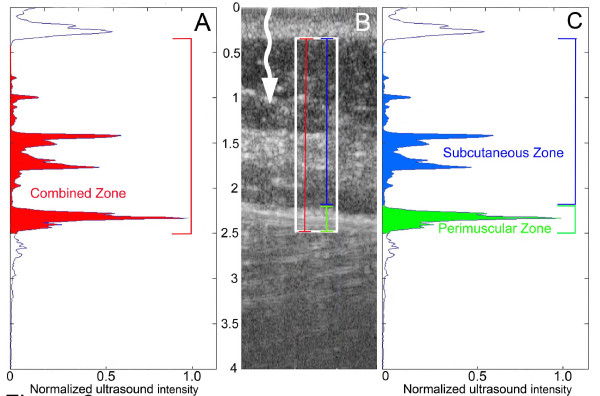
**Ultrasound image analysis method**. B: ultrasound image showing ROI (white box) and whisker lines corresponding to thickness of combined (red), subcutaneous (blue) and perimuscular (green) zones. Units on y axis represent cm. Arrow shows direction of ultrasound beam. A, C: ultrasound intensity profile corresponding to image in B. Colored areas highlight the area under the curve (used as measure of echogenicity) for combined (A, red area), perimuscular (C, green area) and subcutaneous (C, blue area) zones.

The ultrasound radio-frequency raw-data of echoes for each image were imported into Matlab software (The MathWorks, Natick, MA) and converted to a B-scan image using a Hilbert transformation without additional image enhancement. The region of interest (ROI) used for image data analysis was a 1 cm-wide region centered on the middle of the image and located between the deep border of the dermis and the superficial border of the muscle (white box in Figure 2B). For all 64 vertical ultrasound B-scan data lines, grey level profiles within the ROI were identified as a function of the vertical position *z *as *f*_1_*(z), f*_2_*(z)...f*_64_*(z)*. The mean grey level of the 64 data lines at depth *z *was then calculated by . In order to minimize possible variation due to differences in focal depth in different subjects, the mean grey level *f(z)*_*mean *_was normalized to the maximum mean *f(z*_*m*_*) *within the ROI as *f*(*z*)_*mean*_/*f*(*z*_*m*_). Echogenicity was defined as the extended area under the *f(z)*_*mean *_profile curve.

### Subcutaneous and perimuscular zone measurements

Thickness measurements were performed on the acquired ultrasound images by a blinded investigator (inter-rater reliability was greater than 0.98 for all subjective measurements made on ultrasound images--see statistical methods below). All thickness measurements were made in the direction perpendicular to the skin. Combined subcutaneous and perimuscular zone thickness was measured as the distance between the deep border of the dermis and the superficial border of the muscle (Figure 2B, red line). Perimuscular zone thickness was measured as the thickness of the echogenic layered structure within the ROI located closest to the muscle and separated from the nearest, more superficial echogenic layer by more than 2 mm (Figure 2B green line). Subcutaneous zone thickness was measured as the thickness of the zone between the dermis and the superficial border of the perimuscular zone (Figure 2B blue line). Echogenicity for the combined subcutaneous and perimuscular zone was calculated as the area under the curve for the total ROI (Figure 2A). Echogenicity for individual subcutaneous and perimuscular zones was the area under the curve within the portion of the ROI delineated by the respective thickness measurements (Figure 2C). All outcome measures were calculated for individual images (obtained on the subjects' right and left sides) as well as averaged across sides within subjects.

### Statistical methods

T-tests and chi square tests were used to compare LBP and No-LBP groups for subject characteristics. Analyses of covariance using BMI as a covariate, were performed to compare connective tissue thickness and echogenicity of the LBP and No-LBP groups. Although BMI did not differ significantly between LBP and no-LBP groups, individual subject's BMI did explain a significant portion of the variability in each of his/her connective tissue outcome measures. Additionally, univariate correlation analyses based on Pearson's r were used to examine the relationship between indices of symptom severity and disability (McGill pain questionnaire [number of words circled], duration of pain [years], current pain intensity [0-10 scale], exacerbation intensity [0-10 scale], exacerbation frequency [categorized as yearly, monthly, weekly or daily], exacerbation duration [days], percent initial injury and Oswestry disability scale [categorized as mild, moderate or severe] and ultrasound outcomes within subjects with LBP. Statistical analyses were performed using SAS Version 9.1 (SAS Institute, Cary, NC). The outcome measures presented are the averages of right and left sides since no findings were found to be side-specific. Significance levels were determined based on α = .05. In a subset of subjects, estimates of reliability between two blinded investigators were computed based on intraclass correlation coefficients for locating the deep border of dermis, superficial border of muscle and boundary between superficial and perimuscular zones.

## Results

**Figure 3:**
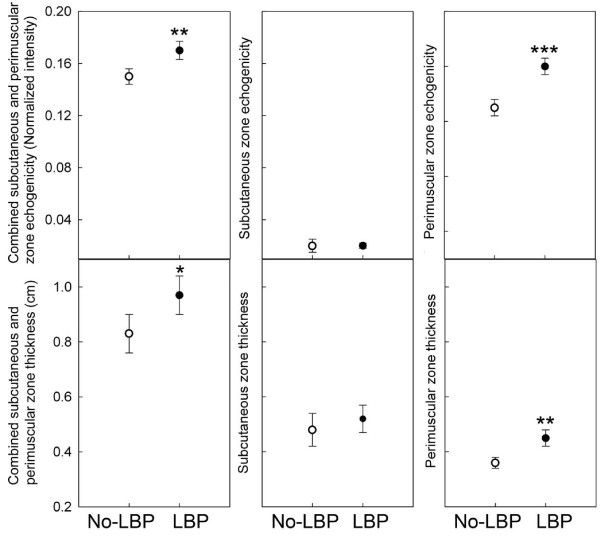
**Echogenicity and thickness measurements for combined, subcutaneous and perimuscular zones**. Open circles indicate No-LBP group and closed circles indicate LBP group; * p < 0.5, **p < .01, ***p < .001, ANCOVA adjusted for BMI (N = 107).

Subject characteristics for LBP and No-LBP groups are shown in Table 1. There were no significant differences between groups in sex (p = .93), mean age (p = .72), mean BMI (p = .81) or physical activity levels (p = .87). However, BMI was highly correlated with perimuscular connective tissue thickness and echogenicity (r = 0.66, p < 0.001, r = 0.51, p < 0.001 respectively). Thus analyses of covariance were performed to examine differences in these outcome measures between LBP and No-LBP groups while adjusting for BMI. Thickness and echogenicity for the combined subcutaneous and perimuscular zone were significantly greater in the LBP group compared with the No-LBP group (ANCOVA, p < .05 and p < .01 respectively) (Figure 3). These differences were primarily due to significantly greater thickness and echogenicity in tissues closer to muscle (perimuscular zone) (ANCOVA, p < .01 and 0 < .001 respectively) but not tissues closer to skin (subcutaneous zone) (ANCOVA, p = .57 and p = .22 respectively) (Figure 3).

**Table 1:** Subject characteristics for LBP and No-LBP groups

	No-LBP (N = 47)	LBP (N = 60)	P value
**Gender**			
Male/Female (%)	43%/57%	42%/58%	0.93
**Age (years)**	39.3 ± 14.1	38.3 ± 13.3	0.72
**BMI (units)**	25.9 ± 0.7	25.7 ± 0.6	0.81
Physical activity level (%)*			0.87
High	67%	62%	
Moderate	26%	29%	
Sedentary	8%	9%	

Values represent Mean ± SD unless otherwise indicated.

*Activity levels based on subjects that completed the survey (No-LBP N = 38, LBP N = 52).

Symptom characteristics of the LBP group are shown in Table 2. There were no significant correlations between ultrasound outcome measures and responses to Mc Gill pain questionnaire, current pain intensity, exacerbation intensity, exacerbation frequency, exacerbation duration, percent initial injury and Oswestry disability scale. However, pain duration was weakly correlated with perimuscular zone thickness (r = 0.35, p = 0.01) and echogenicity (r = 0.30, p = .03) among the LBP group. This relationship persisted after controlling for the influence of BMI.

**Table 2:** Indices of symptom severity and disability in subjects with LBP

**McGill pain questionnaire** (# of words circled) (N = 49)	8.8 ± 4.9
**Duration of pain **(years) (N = 52)	10.0 ± 7.1
**Current pain intensity **(0-10 scale) (N = 54)	3.2 ± 2.2
**Exacerbation intensity **(0-10 scale) (N = 51)	6.1 ± 2.2
**Exacerbation frequency **(N = 52)	
Yearly	12%
Monthly	29%
Weekly	30%
Daily	29%
**Exacerbation duration **(days) (N = 51)	24.1 ± 86.1
**Percent initial injury** (N = 52)	42%
**Oswestry disability scale **(N = 51)	
Mild	67%
Moderate	21%
Severe	12%

Results expressed as Mean ± SD or percentage of subjects surveyed. N = number of subjects that completed each questionnaire.

## Discussion

Human subjects with LBP had, on average, 25% greater perimuscular connective tissue thickness and ultrasound echogenicity in the lumbar region than did subjects without LBP after adjusting for BMI. There was no significant correlation between ultrasound structure and age and no significant differences between men and women. Although we did find a positive correlation between connective tissue structural measures and BMI, this relationship did not explain the differences observed between LBP and No-LBP groups. Moreover, activity levels were similar in both groups suggesting that abnormal connective tissue structure was not due a more sedentary lifestyle in the LBP group.

We do not know, at this point, whether the observed increase in connective tissue thickness and echogenicity are part of the cause or the effect of low back pain. One possibility is that abnormal connective tissue structure (possibly genetically determined) predisposes to the development of chronic or recurrent LBP. Connective tissue also may have remodeled over time in responses to repetitive stresses created by pre-existing altered movement pattern secondary to repetitive motion, habitual postures or sports, with or without tissue injury that predated the onset of LBP [37]. Once pain is present, connective tissue abnormalities may worsen as a result of altered movement patterns due to pain or fear of pain [28]. This would be consistent with the observed positive correlation between connective tissue measurements and symptom duration. In other types of connective tissues (e.g., ligament, joint capsules) the combination of inflammation and reduced mobility can lead to debilitating tissue fibrosis, adhesions and contractures leading to further tissue stiffness and movement impairment [38-45]. So far, however, a similar pathophysiological process involving non-articular connective tissues has not been studied in relation to LBP.

The absence of association between our ultrasound measurements and symptom severity could be due to the fact that pain and disability levels were, on average, quite low in our LBP population, which could have interfered with our ability to detect a correlation. The subjects with low back pain who volunteered for our study were generally active people who managed to live busy lives despite the presence of low back pain, and most of these subjects had mild or moderate levels of pain. Many subjects with and without low back pain practiced yoga or did stretching exercises regularly. Additionally, a number of people with more severe low back pain had to be excluded because of a history of either prior back surgery or corticosteroid injection. Therefore, given these factors, it is remarkable that we found substantial differences in connective tissue morphology between our groups despite the low level of pain in the low back pain group. We might have seen even greater differences between groups and a stronger association with symptoms had our subjects with LBP been more severely impaired and less physically active.

**Figure 4:**
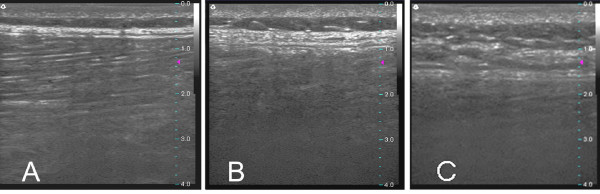
**Examples of ultrasound images illustrating thin (A), thick (B) and multilayered (C) perimuscular connective tissue morphology**.

We have chosen not to use the terms "superficial fascia" and "deep fascia" in describing our findings because of frequent uncertainty in determining the anatomical boundary between these two structures in both live imaging and gross anatomical specimens. According to the traditional anatomical definition, the term "superficial fascia" refers to an enveloping layer directly beneath the skin formed by a fine three-dimensional meshwork containing loose areolar connective tissue and fat within its meshes, while the term "deep fascia" refers to a continuous layer of dense connective tissue that invests muscles and tendons [46]. However, in many subjects we observed multiple echo-rich layers (containing mainly dense connective tissue) separated by echo-poor layers (containing mainly fat) from dermis to muscle, with no clear distinction between superficial and deep fascia (Figure 4C). In other subjects, only one thickened dense connective tissue layer appeared to be present overlying the muscle (Figure 4B). It is thus not clear whether connective tissue morphology such as that shown in Figure 4C is due to the presence of multiple connective tissue layers within the superficial fascia or due to fatty infiltration and disorganization of the perimuscular layer. We believe that the greater average perimuscular connective tissue thickness and echogenicity in the LBP group likely reflects a combination of increased thickness and increased numbers of connective tissue layers in subjects with LBP.

Although this has not yet been studied, an important function of loose connective tissue layers may be to allow dense connective tissue sheets to glide past one another. Increased thickness and disorganization of connective tissue layers due to inflammation, fatty infiltration, fibrosis and adhesions may impair the normal relative movement of connective tissue planes, increase tissue stiffness, decrease range of motion and predispose to further injury [28]. Fatty infiltration and herniation through disorganized connective tissue layers may also cause pain due to trapping of sensory nerve fibers through the collagen matrix [17,21,22].

In this, study, we chose to recruit subjects with chronic LBP of more than twelve months duration in order to maximize our chance of detecting any connective tissue abnormalities, since connective tissue remodeling and fibrosis are relatively slow biological processes that cause morphologically detectable changes over weeks to months, rather than hours to days. We cannot at this point generalize our conclusions to subjects who have had low back pain for less than twelve months. A future longitudinal study evaluating connective tissue in subjects presenting with their first episode of low back and following these subjects over time may shed some light on this issue.

## Conclusion

This study is the first report of abnormal connective tissue structure in the back in a group of human subjects with chronic or recurrent LBP. Increased thickness and disorganization of connective tissue layers may be an important and so far neglected factor in human LBP pathophysiology.

## Competing interests

Helene M. Langevin is a partner of Stromatec, Inc. The following measures were taken to reduce the possibility, or the appearance of any possible conflict of interest: 1) a research assistant with no financial interests in the company monitored all data entry and data integrity as well as handled all regulatory documents; 2) a research engineer, designed programs to automate data collection; 3) any data collection that was not automated and could have a subjective element was performed by blinded technicians and 4) a biostatistician with no financial interests in the company performed all statistical analyses.

## Authors' contributions

HML conceived the study, participated in study design and human subject testing and drafted the manuscript; DST conducted the human subject testing; JRF developed and implemented the ultrasound analysis methods; GJB performed statistical analyses; NAB participated in subjects testing and manuscript preparation; MHK participated in study design; JW assisted in ultrasound data analysis; SMH participated in study design. All authors read and approved the final manuscript.

## Pre-publication history

The pre-publication history for this paper can be accessed here:

http://www.biomedcentral.com/1471-2474/10/151/prepub
